# Preferential and sustained platelet activation in COVID-19 survivors with mental disorders

**DOI:** 10.1038/s41598-024-64094-5

**Published:** 2024-07-12

**Authors:** Norma Maugeri, Rebecca De Lorenzo, Mario Gennaro Mazza, Mariagrazia Palladini, Fabio Ciceri, Patrizia Rovere-Querini, Angelo A. Manfredi, Francesco Benedetti

**Affiliations:** 1 Vita-Salute San Raffaele University, Milan, Italy; 2 Division of Immunology, Transplantation and Infectious Diseases, IRCCS San Raffaele Scientific Institute, Via Olgettina 58, 20132 Milan, Italy; 3 Psychiatry & Clinical Psychobiology, Division of Neuroscience, IRCCS San Raffaele Scientific Institute, Milan, Italy; 4 Hematology and Bone Marrow Transplant Unit, IRCCS San Raffaele Scientific Institute, Milan, Italy

**Keywords:** Mood disorders, Platelet activation, COVID-19, Cell biology, Immunology, Biomarkers, Neurology

## Abstract

Pre-existing mental disorders are considered a risk factor for severe COVID-19 outcomes, possibly because of higher vascular burden. Moreover, an unconventional platelet activation characterizes COVID-19 and contributes to inflammatory and thrombotic manifestations. In the light of the inflammation theory of mental disorders, we hypothesized that patients with mental disorders could be sensitive to the SARS-CoV-2 elicited platelet activation. We investigated platelet activation in 141 COVID-19 survivors at one month after clearance of the virus, comparing subjects with or without an established pre-existing diagnosis of mental disorder according to the DSM-5. We found that platelets from patients with a positive history of psychiatric disorder underwent unconventional activation more frequently than conventional activation or no activation at all. Such preferential activation was not detected when platelets from patients without a previous psychiatric diagnosis were studied. When testing the effects of age, sex, and psychiatric history on the platelet activation, GLZM multivariate analysis confirmed the significant effect of diagnosis only. These findings suggest a preferential platelet activation during acute COVID-19 in patients with a pre-existing psychiatric disorder, mediated by mechanisms associated with thromboinflammation. This event could have contributed to the higher risk of severe outcome in the psychiatric population.

## Introduction

Patients with severe mental illness (SMI) suffered a tremendous death toll from COVID-19 pandemic. In a large systematic review and meta-analysis, including 1,469,731 patients with COVID-19, of whom 43,938 had mental disorders, the odds ratio for COVID-19 mortality was estimated at 2.00 for any mental disorder, at 1.99 for mood disorders, and at 2.23 for exposure to antidepressants^1^. Similar figures were then confirmed by further large meta-analytical^2,3^ and prospective cohort studies^4,5^.

It is not clear which mechanisms could underpin the increased risk COVID-19 severe outcome and death in patients with mood disorders. A sparse, but consistent evidence associated mental disorders with atypical responses to pathogens, hypothesizing a role for infections in the emergence of mental illness^6–8^. The study of post-acute COVID-19 sequelae showed a high prevalence of psychopathological symptoms and neurocognitive impairment in survivors, predicted by systemic inflammation as observed at the onset and in the months following the clearance of the virus^9–12^. Independent of reported infections and of active somatic immune diseases, immune activation and inflammation have been observed in mood disorders^13^. According to the inflammation theory of mood disorders, the deregulation of the immune system involves elevated peripheral inflammatory markers, activation of microglia into the brain, and higher monocyte/macrophage expression of genes regulating inflammation and coagulation^14^. Consistent evidence also associated inflammation with endothelial dysfunction and platelet activation in mood disorders, also leading to higher morbidity and mortality for cardiovascular disease^15–17^.

P-selectin and HMGB1 expression, the loss of von Willebrand factor (VWF) content as well as the release of extracellular vesicles (EVs) occur during platelet activation^18^. In patients hospitalized during the ‘first wave’ in March-June 2020, we and others have observed a widespread activation of platelets from patients with COVID-19^19–22^, as reflected by the higher fraction of platelets expressed of P-selectin and of the prototype endogenous inflammatory signal, HMGB1 and by the accumulation in the plasma of platelet-derived (EV) expressing HMGB1. Notably, the concentration of HMGB1+platelet EV correlated with inflammatory markers, with the activation of the coagulation cascade and with the patients prognosis^22^. We also observed that platelet activation was heterogeneous and followed a bimodal distribution. A fraction of the patients had platelets that upregulated the expression on the surface of P-selectin and HMGB1 and conversely had a low content of VWF. In an equally large group of patients the activated platelets had low surface P-selectin because of accelerated proteolysis^18,22^. The latter event involved the cross-talk of SARS-CoV-2 with CD147 expressed on platelets^22^. Notably, platelet activation triggered by SARS-CoV-2 infection in vitro replicates alpha and dense granule content depletion, HMGB1 membrane localization, and accelerated proteolysis of P-selectin. This response is readily provoked by viral isolates, pseudoviruses, or recombinant Spike protein isoforms, while attempts to replicate these phenomena using potent platelet agonists have proven unsuccessful, highlighting the unique nature of SARS-CoV-2-induced platelet activation^18,22^. This uniqueness may stem from the involvement of different receptors, such as the CD147 molecule.

The CD147 receptor is the main upstream stimulator of matrix metalloproteinases^23^, which are upregulated in many psychopathological conditions, including mood disorders, schizophrenia, addiction, and stress- and fear-related conditions, also being considered as a putative target for treatment^24–27^. CD147 is expressed in neurons, and was suggested to play a role in the neuropathological consequences of COVID-19^28^. Though CD147 expression was never studied in mental illness, in animal models psychological stress up-regulated CD147 expression through the increased release of norepinephrine and the activation of the Beta-Arrestin1/ERK pathway, a mechanism implicated in the detrimental effects that stress exerts on occurrence and prognosis of malignant tumors^29^, and suggested to play a role in enhancing susceptibility to SARS-CoV-2 infection^30^. Furthermore, another signal associated with relentless platelet activation in COVID-19, the prototype alarmin/DAMP HMGB1 promotes neuroinflammation, acting on microglia via the HMGB1/STAT3/p65 axis and autophagy^31,32^ while causing fatigue in patients with inflammatory bowel disease alone or by amplifying pathways associated with the IL-1 beta response^33^.

We hypothesized that patients with mental disorders could be particularly sensitive to the SARS-CoV-2 elicited, CD147-mediated platelet activation. We tested this hypothesis in COVID-19 survivors, comparing subjects with or without an established diagnosis of mental disorder previous to SARS-CoV-2 infection.

## Results

The clinical and demographic characteristics of the participants, categorized according to personal history of psychiatric disorder prior to COVID-19 and platelet activation, are summarized in Table 1. No between-group differences were observed in age, length of stay, laboratory tests at the time of hospital admission, and cardiovascular comorbidities, with the exception of dyslipidemia and cerebrovascular disease that had nevertheless a low prevalence in both groups (Table 1). Sex distributed unevenly among groups: in agreement with the epidemiology of mood and anxiety disorders and previous studies in COVID-19 survivors, patients with a positive history of psychiatric disorder showed a 2.2:1 female/male ratio, while patients with a negative history showed an opposite 3:1 male/female ratio (χ^2^ = 20.09, d.f. 1, *p* < 0.0001).

**Table 1:** Clinical and demographic characteristics of participants.

	Positive history of psychiatric disorder (n = 35)			Negative history of psychiatric disorder (n = 106)			F or χ^2^	*p*
	Not activated	Classical activation	Alternative activation	Not activated	Classical activation	Alternative activation		
N (% within group)	8/35 (22.9)	6/35 (17.1)	21/35 (60)	42/106 (39.6)	26/106 (24.5)	38/106 (35.8)	282.00	< 0.0001
Sex (M/F)	2/6	3/3	6/15	29/13	20/6	29/9	21.81	0.0006
Age	57.50 ± 14.90	54.33 ± 13.04	57.70 ± 12.41	52.60 ± 13.26	57.85 ± 13.79	58.18 ± 16.47	0.833	0.528
Obesity	4/4	3/3	12/9	29/13	17/9	26/12	8.641	0.279
T2DM	0/8	0/6	1/20	3/39	6/20	5/33	11.989	0.101
Dyslipidemia	1/7	1/5	2/19	13/29	2/24	7/31	16.763	**0.019**
Peripheral vasculopathy	1/7	0/6	1/20	4/38	1/25	2/36	9.986	0.194
Cerebrovascular disease	0/8	0/6	3/18	1/41	1/25	1/37	17.818	**0.013**
Arterial hypertension	4/4	3/3	5/16	13/29	11/15	17/21	11.379	0.123
Duration of hospitalization (days)	10.71 ± 7.41	3.83 ± 4.02	1026 ± 7.83	15.79 ± 15.54	10.64 ± 9.77	12.64 ± 10.38	1.558	0.178
Time after discharge (days)	32.25 ± 10.11	26.00 ± 8.79	27.11 ± 12.03	32.00 ± 16.10	25.17 ± 8.68	27.91 ± 10.18	1.221	0.303
Laboratory data at hospital admission								
CRP	53.74 ± 82.06	54.45 ± 60.43	63.04 ± 77.37	64.36 ± 65.67	85.06 ± 80.47	54.19 ± 51.08	0.689	0.633
Neutrophils	3.99 ± 2.34	3.10 ± 1.16	4.95 ± 3.09	5.10 ± 2.30	5.29 ± 2.67	5.67 ± 2.65	1.155	0.336
Lymphocytes	1.11 ± 0.19	0.74 ± 0.34	1.12 ± 0.66	1.27 ± 0.65	1.18 ± 0.61	1.24 ± 0.76	0.680	0.639
Platelets	226.71 ± 96.72	217.67 ± 55.11	245.75 ± 123.25	237.35 ± 107.34	229.91 ± 81.39	201.63 ± 64.81	0.695	0.629
NLR	3.74 ± 2.46	4.37 ± 1.26	5.76 ± 4.53	5.70 ± 6.08	5.53 ± 4.01	7.12 ± 7.56	0.566	0.726
PLR	211.18 ± 104.99	321.17 ± 115.06	250.18 ± 140.78	347.38 ± 202.25	228.36 ± 115.54	218.44 ± 191.99	0.406	0.844
SII	1019.47 ± 1163.84	966.25 ± 512.93	1451.94 ± 1728.85	1250.36 ± 977.47	1239.55 ± 969.46	1275.05 ± 1543.85	0.189	0.966

Significant values are in bold.

Divided according to personal history of psychiatric disorder preceding COVID-19, to platelet activation and level of significance of the observed differences.

*CRP* C-reactive protein, *NLR* neutrophils to lymphocyte ratio, *PLR* platelet to lymphocyte ratio, *SII* Systemic Inflammation Index, *T2DM* type 2 diabetes mellitus. Statistical values refer to one-way ANOVAs or χ^2^ tests, as appropriate.

91/141 patients (64.5%) had circulating activated platelets at the time of analysis, 28.65 ± 12.28 days after discharge from the hospital. This finding indicates that stimuli that support platelet activation persist in COVID-19 survivors even after clinical recovery and normalization of the molecular assays for SARS-CoV-2 infection. According on the characteristics of platelets, we observed that (i) a group 32 patients had classically activated platelets, with high surface P-selectin and HMGB1 expression and depleted alpha granules, as revealed by the low VWF content; (ii) a group of 59 patients had unconventional platelet activation with low P-selectin expression, high surface expression of HMGB1 and the virtually complete depletion of alpha granules.

The extent of activation was higher in patients with (27/35, 77.1%) than without (64/106, 60.4%) a previous psychiatric history. Moreover, the type of activation was unevenly distributed among patients with or without mental illness (Fig. 2; χ^2^ = 6.386, d.f. 2, *p* = 0.0411). Patients with a positive personal history of psychiatric disorder had a significantly higher prevalence of unconventional activation (21/35, 60%) in respect to classical activation (6/35, 17.1%) or lack of platelet activation (8/35, 22.9%). Patients without a previous psychiatric diagnosis showed a slight prevalence of quiescent platelets (42/106, 39.6%) compared to those activated following a classic (26/106, 24.5%) or unconventional pattern (38/106, 35.8%). Sex was not associated with activation (χ^2^ = 1.292, d.f. 2, *p* = 0.524).

When testing the effects of age, sex, and previous psychiatric history on platelet activation in a multivariate analysis, the GLZM analysis selected a model containing DSM 5 diagnosis alone as the best predictor (AIC = 303.04, LR χ^2^ = 6.352, *p* = 0.0418). When considering together the classical and unconventional platelet activation (or activated vs not activated platelets), a GLZM analysis of homogeneity of variances confirmed the significant effect of diagnosis in enhancing the rate of activation (χ^2^ = 4.287, *p* = 0.0384), with non-significant effects of sex and age. When separating groups in three categories (non activated, classically activated, and unconventionally activated) the GLZM analysis again confirmed the significant effect of diagnosis only (χ^2^ = 6.113, *p* = 0.0471), with non-significant effects of sex and age. When stratifying patients according to a diagnosis of Mood Disorder (MDD or BD) or of Anxiety Disorder, no difference in the distribution of platelet activation was observed between the two DSM-5 categories.

## Methods

We studied 141 COVID-19 survivors, 28.65 ± 12.28 days after clearance of the virus (negative real-time reverse-transcriptase polymerase chain reaction from a nasopharyngeal and/or throat swab for COVID-19) and hospital discharge (April–May 2020), in a naturalistic setting during an ongoing prospective cohort study at IRCCS San Raffaele Hospital in Milan (Covid-BioB, ClinicalTrials.gov NCT04318366). The study conforms to the declaration of Helsinki and obtained ethical approval from the Institutional Review Board of IRCCS San Raffaele Scientific Institute (protocol number 34/int/2020). Diagnosis of COVID-19 was made after clinical and radiological findings suggestive of COVID-19 pneumonia at the admission to the Emergency Department, as confirmed by positive real-time reverse-transcriptase polymerase chain reaction from a nasopharyngeal and/or throat swab. Written informed consent was obtained from all participants. An unstructured clinical interview was conducted by well-trained psychiatrists in charge using the best estimation procedure, taking into account available charts, computerized medical records, and, if needed, the information provided by a relative. Sociodemographic and clinical data were collected using a data extraction form, including age, sex, and psychiatric history. A previous positive psychiatric history was defined by the need of previous contacts with psychiatrists and/or psychotherapists, and need for treatment.

Agreement on previous diagnosis (DSM 5 criteria) was reached among 2 psychiatrists (FB and MGM), the patients, and their relatives, exploiting all available sources of information. 35/141 patients (24.8%) had received a previous diagnosis of a DSM 5 disorder: Major depressive Disorders (MDD, n = 15); Bipolar Disorder (BD, n = 2); Panic Disorder (n = 8); Social Anxiety (n = 4); Generalized Anxiety Disorder (n = 6). Patients were then grouped has having a Mood Disorder (n = 17) or an Anxiety Disorder (n = 18). No patient was taking psychiatric drugs when studied.

**Figure 1:**
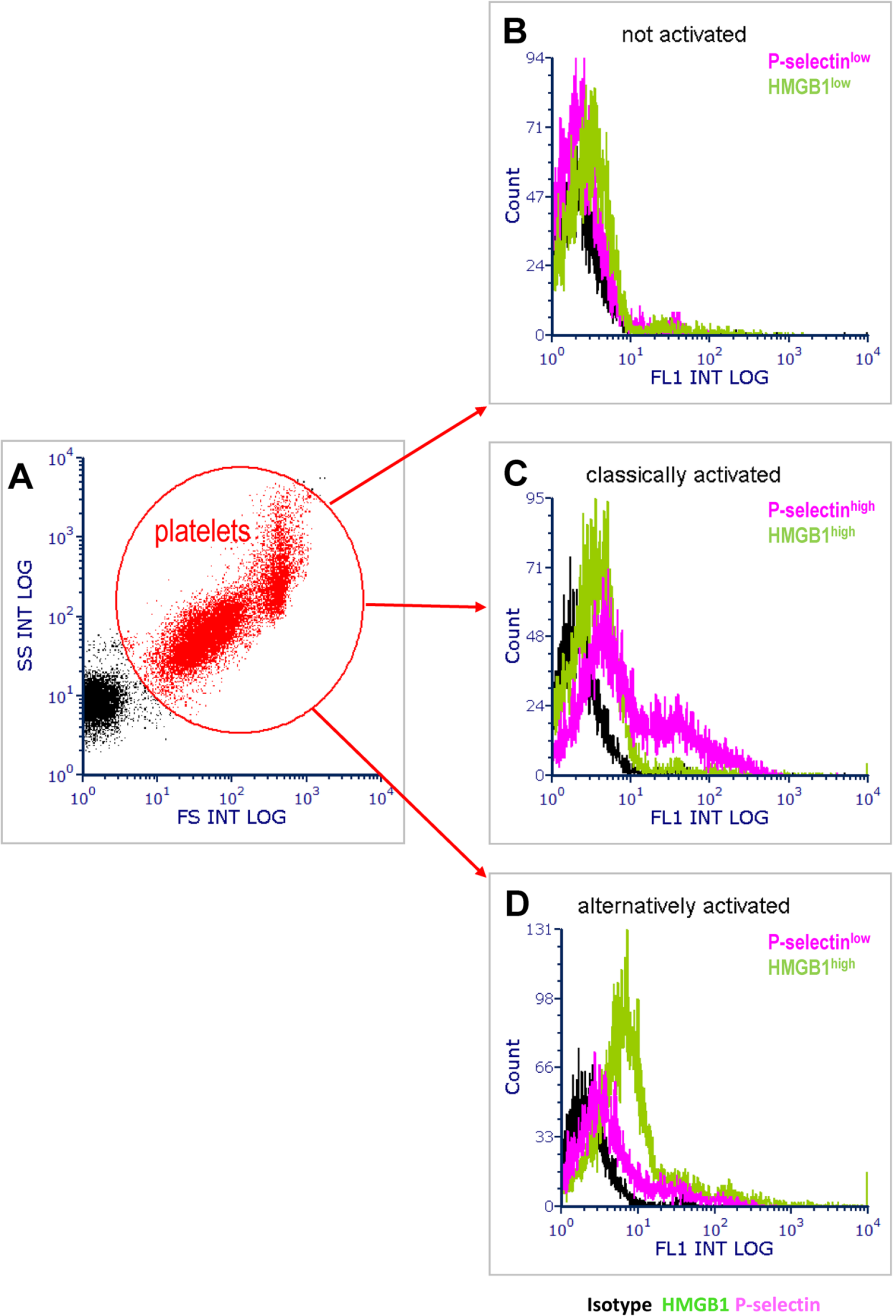
Gating strategy used for analysis by flow cytometry of platelets. Platelets were identified within the CD61^+^ population (threshold of acquisition) based on their side scatter (y axis) and forward scatter (x axis) characteristics (**A**). The expression of P-selectin (pink) and HMGB1 (green) was analyzed. The histograms represent non-activated (**B**), classically activated (**C**) and unconventional activated (**D**) platelets.

Blood sampling and platelet analysis. Patients were studied one month after discharge. Venous blood was drawn through a 19-gauge butterfly needle. After discarding the first 3–5 mL, blood was carefully collected in tubes containing Na_2_ EDTA and immediately fixed (Thrombofix, Beckman Coulter Italy). Samples were then stored at 4 °C. To determine platelet activation, they were labelled with monoclonal antibodies (mAbs) against CD61 (Platelet GPIIIa, clone SZ21, Beckman Coulter), against P-selectin (CD62P, clone Thromb‐6, Beckman Coulter), and against HMGB1 (clone 3E8, Biolegend). To determine the VWF platelet content, samples were labelled with mAbs anti CD61, permeabilized with Fix&Perm (Caltag) and incubated with the anti-VWF mAb (4F9). Platelets were identified within the CD61^+^ population (threshold of acquisition) by their side scatter and forward scatter characteristics. Samples incubated with irrelevant isotype-matched IgGs served as controls. Samples were analyzed on a daily aligned Navios flow cytometer (Beckman Coulter, Milan, Italy)^22,34^. The gating strategy is depicted in Fig. 1.

**Figure 2:**
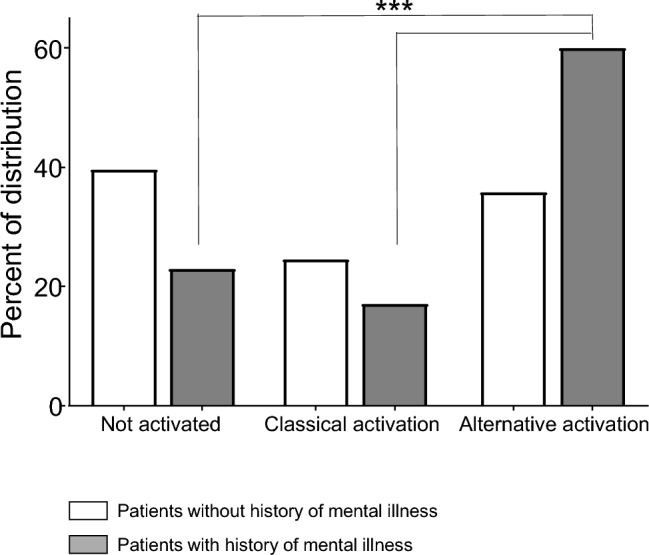
Percent distribution of platelet activation in participants with or without a previous history of mental illness. *: χ^2^ = 6.386, d.f. 2, *p* = 0.0411.

To test the effect of predictors on outcomes, and considering the a priori expected significant interaction of independent factors (age, sex, diagnosis) and the possible violation of parametric assumptions (distribution of variables, homogeneity of variances), independent variables were entered into a Generalized Linear Model (GLZM) analysis of homogeneity of variances to test for the main effects and for the interaction of continuous and categorical variables, with a logit link function^35^. Parameter estimates were obtained with iterative re-weighted least squares maximum likelihood procedures. The significance of the effects was calculated with the likelihood ratio (LR) statistic, which provides the most asymptotically efficient test known, by performing sequential tests for the effects in the model of the factors on the dependent variable, at each step adding an additional effect into the model contributing to incremental χ^2^ statistic, thus providing a test of the increment in the log-likelihood attributable to each current estimated effect^36,37^. The quality of the statistical models was checked using the entropy maximization principle of the Akaike information criterion (AIC)^38^ (Fig. 2).

## Discussion

This is the first study to report a significantly higher rate of platelet activation after SARS-CoV-2 infection patients with a pre-existing DSM-5 psychiatric diagnosis. This effect was explained by a higher rate of unconventional platelet activation, which in previous studies has been shown to be exquisitely dependent on the CD147 receptor on the platelet surface, and specifically triggered by the SARS-CoV-2 infection^22^.

The integral plasma membrane glycoprotein CD147 (cluster of differentiation 147), also known as basigin, or extracellular matrix metalloproteinase inducer (EMMPRIN), is a member of the immunoglobulin superfamily of proteins that are involved in the recognition, binding, or adhesion processes of cells. CD147 mediates inflammatory activation in several cells, including macrophages, and the expression of inflammatory cytokines and chemokines in endothelial cells, which may contribute to the pathogenesis of inflammatory diseases^39^. CD147 is widely expressed in many cell types, including haematopoietic, epithelial, endothelial cells and leukocytes^40^. In the normal brain, it is expressed in neurons^28^, astrocytes^41^, vascular endothelial cells^42^, microglia^43^, and it is suggested to play a major role in the pathogenetic mechanisms involved in ischemic stroke, brain injury, multiple sclerosis, Alzheimer׳s disease^43^.

Human platelets constitutively express CD147. It is a bona fide activatory receptor for platelets, and has been shown to be important for the platelet /leukocyte interaction^44–47^. CD147 stimulation by SARS-CoV-2 via the spike protein S1 subunit stimulates platelets (monitored by the up-regulation of HMGB1, aggregation, granules discharge, shedding and release of P-selectin and release of platelet HMGB1+EVs), contributing to the early and intense activation of platelets found in COVID-19 patients and associating with fatal outcomes^22^. This event was previously described in subjects with COVID-19 as " unconventional platelet activation" and is reproduced by in vitro activation of the CD147 receptor on platelets by various agonists^22^. Experiments with SARS-COV-2 clinical isolates have suggested that accelerated proteolytic elimination of P-selectin underlies the relative absence of P-selectin on the surface of activated platelets^22^. The observation we report here, that such activation is still substantial well after clinical recovery and viral clearance, indicates that endogenous agonists capable of supporting CD147 activation can be generated even after resolution of the infection. Further studies are needed to identify the molecular pathways involved.

Although CD147 was never studied in psychiatric conditions, indirect evidence suggests its involvement. CD147 is the main stimulator of matrix metalloproteinases, which are key enzymes in maintaining the integrity of the extracellular matrix, and in modulating inflammation and innate immunity, to regulate barrier function, inflammatory cytokine and chemokine activity, and to generate chemokine gradients^48^. Matrix metalloproteinases are upregulated in several psychiatric conditions including MDD and BD^24,25,49^, correlating with anxiety^24^, and thus suggesting a possible upregulation of CD147. Moreover, in animal models of disease stress enhances the expression of CD147^30^, with its upregulation possibly mediating the detrimental effect of the stress hormone norepinephrine on health^29^.

In proposing a mechanism for the enhanced CD147-dependent platelet activation in patients with psychiatric conditions, it is then tempting to hypothesize that it might be due to a higher expression of CD147, or to a higher responsivity of the CD147 signaling pathway, linked with the psychiatric diagnosis, and/or with a higher sensitivity to the effects of stress associated with the pandemic and with the life-threatening experience of COVID-19 pneumonia. This mechanistic hypothesis needs to be tested in further studies.

The dysregulation of neurotransmitters, such as serotonin, plays a critical role in the pathogenesis of various psychiatric conditions. Selective serotonin reuptake inhibitors were developed based on this original hypothesis, proposed in the latter half of the twentieth century, and have demonstrated efficacy in alleviating symptoms in depression patients. However, serotonin is not exclusively produced within the central nervous system; enterochromaffin cells in the periphery also synthesize and store serotonin in granules, which can be released into the peripheral blood upon stimulation. Platelets absorb and store a significant amount of the neurotransmitter in dense granules^50^, that is released when platelets undergo activation, contributing to their biological actions. We have previously shown that release of dense granule content in the environment, is an integral part of the constitutive, unconventional activation of platelets we and others observe in patients with COVID-19. The significantly higher association of this pattern with mental disorders, as described in our study, supports the hypothesis that not only stable levels of serotonin but also rapid variations in systemic serotonin levels due to synchronized platelet activation could influence brain responses. This phenomenon may contribute to shaping disease outcomes, including the risk of developing long-term psychiatric sequelae.

It is interesting to note that the association between platelet activation and psychiatric disorders appears to be more stringent than the association between the overall extent of inflammation in patients with COVID-19, as reflected by systemic plasma levels of cytokines such as TNF-alpha, IL-6, and CXCL-10, or downstream acute phase proteins produced by hepatocytes in response to inflammatory cytokines like C-reactive protein (CRP), even if the latter signals are much better predictors of short-term outcomes^51^.

The same trend is observed for other inflammatory and neuroendocrine markers, such as chitinase-3-like protein-1^52^ or chromogranin A^53,54^. This observation supports the idea of a specific role of platelets in linking extent and characteristics of systemic inflammation with long term effects of COVID-19 in the central nervous system. While our study focused on the association between platelet activation and psychiatric disorders, the lack of significant associations with other markers of inflammation, such as CRP, suggests a unique relationship between platelet activation and psychiatric outcomes in the context of COVID-19.

*Cardiovascular comorbidities are associated with CD147 over-expression*^45^* and influence the life expectancy of patients with mental illness*^55^*. In the subjects we have studied, conventional cardiovascular risk factors did not statistically differ between patients with or without a history of psychiatric disorders, with the exception of dyslipidemia and cerebrovascular diseases, which were however relatively rare* (Table 1)*. Further studies are required to gain deeper insights into the potential influence of comorbid cardiovascular risk factors on clinical outcomes.*

Moreover, further studies in appropriate ad hoc experimental models are necessary to investigate this possibility and elucidate the underlying mechanisms. These studies could provide valuable insights into the specific pathways through which platelets contribute to the development of psychiatric sequelae in patients with COVID-19 and other inflammatory conditions.

In conclusion, our present findings reveal unconventional platelet activation in patients with a DSM-5 diagnosis of psychiatric disorder, mediated by mechanisms robustly associated with activation of the coagulation cascade, that could have contributed to the worse rates of fatal outcomes observed during the acute phase of the illness. Beyond COVID, this observation warrants interest to explore a potential new mechanism and target for treatment, linking endothelial dysfunction, platelet activation, and inflammation in psychiatric conditions, which are a focus of clinical and research interest associated to higher morbidity and mortality for cardiovascular disease^15–17^.

Strengths of the present study include a focused research question and a real-world experimental setting, but our results must be viewed in light of limitations. No patient was drug-naive, and the drug treatments administered during the illness could have influenced the clinical and biological picture. Recruitment was in a single center and in a single ethnic group, thus raising the possibility of population stratifications and limiting generalizability.

Our study enrolled consecutive patients admitted to the COVID-19 wards of our institution, as detailed in Rovere-Querini et al.^56^. The proportion of patients with psychiatric diagnoses in our study mirrors the prevalence of psychiatric and cardiovascular comorbidities observed among patients presenting at our institution's emergency department during the specified period. Accordingly, approximately 20–30% of the general population experiences psychiatric disorders, primarily anxiety and depression^12,57,58^. The male-to-female ratio among patients also aligns well with the anticipated ratio. However, the limited number of patients with psychiatric diagnoses, particularly in the depression and anxiety disorder groups, relative to the overall sample size represents a potential constraint and we acknowledge the need for cautious interpretation of our findings. We recognize the need for further adequately powered studies to elucidate comprehensively the relationship between COVID-19 and psychiatric conditions. In particular, given the relevance of platelet activation in patients with mood disorders and psychosis, which has been implicated in their enhanced risk of cardiovascular events, and the potential effects of antidepressants or antipsychotic agents on platelet function we are investigating the effects of COVID-19 in an ongoing prospective study.

These limitations, however, do not bias the main finding of a higher rate of persistent CD147-dependent platelet activation in COVID-19 survivors with mental illness.

## Data Availability

The data underlying this article will be shared on reasonable request to the corresponding author.
